# *epihet* for intra-tumoral epigenetic heterogeneity analysis and visualization

**DOI:** 10.1038/s41598-020-79627-x

**Published:** 2021-01-11

**Authors:** Xiaowen Chen, Haitham Ashoor, Ryan Musich, Jiahui Wang, Mingsheng Zhang, Chao Zhang, Mingyang Lu, Sheng Li

**Affiliations:** 1 The Jackson Laboratory for Genomic Medicine, 10 Discovery Drive, Farmington, CT 06032-2374 USA; 2 Weill Cornell Medicine, New York, NY USA; 3 The Jackson Laboratory for Mammalian Genetics, Bar Harbor, ME USA; 4 The Jackson Laboratory Cancer Center, Bar Harbor, ME USA; 5 Department of Genetics and Genome Sciences, University of Connecticut School of Medicine, Farmington, CT USA; 6 Department of Computer Science and Engineering, University of Connecticut, Storrs, CT USA

**Keywords:** Tumour heterogeneity, Software

## Abstract

Intra-tumoral epigenetic heterogeneity is an indicator of tumor population fitness and is linked to the deregulation of transcription. However, there is no published computational tool to automate the measurement of intra-tumoral epigenetic allelic heterogeneity. We developed an R/Bioconductor package, *epihet*, to calculate the intra-tumoral epigenetic heterogeneity and to perform differential epigenetic heterogeneity analysis. Furthermore, *epihet* can implement a biological network analysis workflow for transforming cancer-specific differential epigenetic heterogeneity loci into cancer-related biological function and clinical biomarkers. Finally, we demonstrated *epihet* utility on acute myeloid leukemia. We found statistically significant differential epigenetic heterogeneity (DEH) loci compared to normal controls and constructed co-epigenetic heterogeneity network and modules. *epihet* is available at https://bioconductor.org/packages/release/bioc/html/epihet.html.

## Introduction

DNA methylation is a critical epigenetic modification. Aberrant DNA methylation is a hallmark of cancers and plays an important role in the initiation, progression, and manifestation of many cancers^1–3^. Recently, the availability of bisulfite sequencing including whole-genome bisulfite sequencing (WGBS) and reduced-representation bisulfite sequencing (RRBS), enables the quantification of DNA methylation at a single base-pair resolution^4–6^. Bisulfite sequencing reads covering multiple CpGs can profile the phased methylation states (C, ^m^C) for all CpGs in that read. Therefore, bisulfite sequencing provides a powerful tool to assess intra-tumoral cell-to-cell epigenetic variability. Many primary cancers feature with high levels of intra-tumor epigenetic heterogeneity, such as acute myeloid leukemia (AML)^7^, chronic lymphocytic leukemias (CLL)^8^, large diffuse B-cell lymphoma^9^, and Ewing sarcoma tumors^10^. These studies have shown that epigenetic diversification emerges in cancer cell populations and changes during disease progression^7,9^. Epigenetic variation causes neoplastic transformation and fitness^11^. Although some cancers (such as AML) have fewer genetic mutations than most other cancers^12^, higher tumor epigenetic heterogeneity burden is linked with worse clinical outcome in cancer patients^7–9^. In addition, epigenetic allelic heterogeneity is associated with higher levels of transcriptional heterogeneity^7^. Lastly, epigenetic heterogeneity broadly affects the cancer genome. The epigenetic heterogeneity is lower in gene regulatory region (promoters, CGI, exons, enhancers) than intergenic regions, CGI shelves and shores^8^.

**Figure 1:**
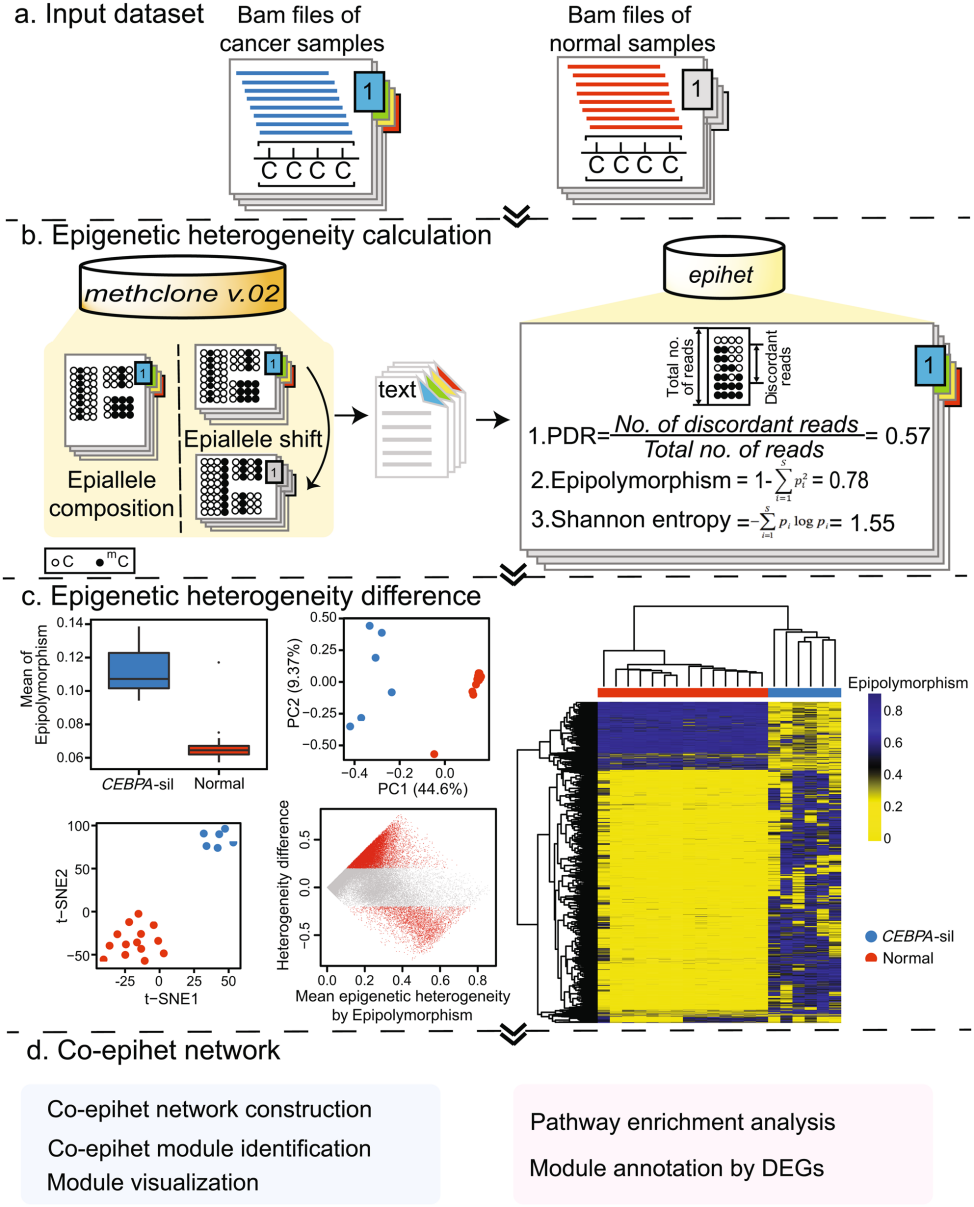
Schema of epigenetic heterogeneity calculation and analysis. (**a**) The Bam files of bisulfite sequencing form the input for *methclone v.02*. (**b**) When the input of *methclone v.02* is one sample’s Bam file, it gives the epiallele composition and the percentage of reads support each of compositions at one locus in the sample. When the input of *methclone v.02* is bam files of two samples, except the above information for both samples, it calculates epiallele shift for each locus between two samples. The output format of *methclone v.02* is a compressed text file. Then, *epihet* takes the compressed text file as the input file and calculates intra-tumoral epigenetic heterogeneity for one sample. Here, four circles on each line represent four adjacent CpG sites, i.e. one locus. (black: methylated CpG; white: unmethylated CpG). (**c**) *epihet* compares epigenetic heterogeneity of different groups and identifies DEH loci using an example from 6 primary AML patients with silenced *CEBPA* gene expression (*CEBPA*-sil) and 14 normal bone marrow CD34^+^ cell samples. Upper left: boxplot shows mean Epipolymorphism of patients with *CEBPA*-sil are higher than normal samples. Upper right: a two-dimensional principal component analysis. Middle: Heatmap of Epipolymorphism values based on the most variant 5% loci across 6 AML patients and 14 normal samples using hierarchical clustering analysis. Lower left: t-SNE visualizations of 6 AML patients and 14 normal samples clustered into two groups. Lower right: MA plot showing the DEH loci between AML patients and normal samples (absolute value of mean epigenetic heterogeneity difference > 0.2, FDR adjusted p-value < 0.05), which are highlighted in red. See also Supplementary Figs. S1 and S2 for PDR and Shannon entropy. (**d**) Co-epihet network construction. A range of network analysis functions are included in *epihet*, including network module identification, module visualization, pathway enrichment analysis for each module, module annotation by DEGs.

Multiple metrics have been developed to evaluate the intra-tumoral epigenetic heterogeneity and dynamics, including global epiallele shift measured by the tool *methclone* that we developed^13^, local disordered reads measured by proportion of discordant reads^8^, epiallele diversity measured by Epipolymorphism^14^ and Shannon entropy^15^. All these metrics consider the phased DNA methylation pattern spanning multiple adjacent CpGs when covered by a single sequencing read as one unit, which is called one locus. Heterogeneous methylation patterns at the given locus range from a complete of un-methylation to full methylation. DNA methylation state at a given locus in a cell population can form a mixture of epigenetic patterns (“epialleles”) with different frequencies. Using these DNA methylation patterns, all of the metrics evaluate intra-tumoral epigenetic heterogeneity through examining epialleles that change their frequencies. To the best of our knowledge, there is no publicly available computational tool to automate the measurement of intra-tumoral epigenetic allelic heterogeneity, differential heterogeneity, functional evaluation, and visualization. To fill this gap, we developed an R/Bioconductor package *epihet* that can automatically calculate and characterize epigenetic heterogeneity based on methylation pattern information from the tool *methclone*. Further, *epihet* can perform differential epigenetic heterogeneity analysis, co-epigenetic heterogeneity (co-epihet) network construction, and visualization of results (Fig. 1).

## Results

### *Epihet* overview

We previously developed a C++-based open source *methclone*, in which epiallele compositional changes of each locus in the genome covered by bisulfite sequencing at two different groups is computed via the combinatorial entropy difference. In this work, we updated *methclone* (https://github.com/TheJacksonLaboratory/Methclone), and introduced *epihet*, an R package to compute intra-tumoral epigenetic heterogeneity and to perform further downstream analysis, which has been accepted by Bioconductor (https://bioconductor.org/packages/release/bioc/html/epihet.html). Firstly, the input of *methclone v.02* is the Bam files of bisulfite sequencing data from cancer or normal samples (Fig. 1a). With *methclone v.02*, the users have the flexibility to input one sample or two samples. When the Bam file of one sample is provided, *methclone v.02* calculates the dominant methylation pattern information of one locus in the sample. When the Bam files of two samples are provided, *methclone v.02* calculates the dominant methylation pattern information of one locus in each sample and the epiallele shift between two samples (Fig. 1b). Furthermore, the users can define two parameters in the process of methylation pattern calculation. The first parameter is the minimum read coverage at a single base of one locus, which is used to filter loci and increase the power of the statistical tests. By default, *methclone v.02* discards loci that have coverage below 60. The selection of this parameter depends on the read depth of the investigated bisulfite sequencing data. The second parameter is maximum distance between first and forth bases of one locus. The default value is 72, which depends on the read length of the investigated bisulfite sequencing data.

### Epigenetic heterogeneity analysis

Based on DNA methylation pattern information obtained from *methclone v.02*, we developed an open-source R package *epihet* to evaluate intra-tumoral epigenetic heterogeneity including proportion of discordant reads (PDR), Epipolymorphism and Shannon entropy (Fig. 1b). Additionally, *epihet* is flexible to add the customized metrics the users develop or interest in. The result of all the samples in *epihet* can be summarized as a measurement matrix with a row for each locus and a column for every sample. The structure of the matrix can enable *epihet* to compare epigenetic heterogeneity difference between cancer and normal samples, including: (1) visualizing the mean epigenetic heterogeneity of samples by different groups using boxplot; (2) performing Principal Component Analysis (PCA); (3) performing hierarchical clustering analysis; (4) performing t-Distributed Stochastic Neighbor Embedding (t-SNE) analysis to understand the relative similarity of epigenetic allele variations among different groups. To demonstrate the package, we applied *epihet* to 6 primary AML patients with silenced *CEBPA* gene expression (*CEBPA*-sil) from Glass et al.^16^ and 14 normal bone marrow (NBM) CD34^+^ cell samples from Li et al.^7^. Methylation profiles of these samples were measured using eRRBS. Here, mean Epipolymorphism of *CEBPA*-silenced patients is higher than normal samples. Multiple clustering analyses show that cancer and normal samples can form distinct clusters based on epiallele heterogeneity (Fig. 1c, see Supplementary Figs. S1a–d and S2a–d for PDR and Shannon entropy).

### Differential epigenetic heterogeneity analysis

**Figure 2:**
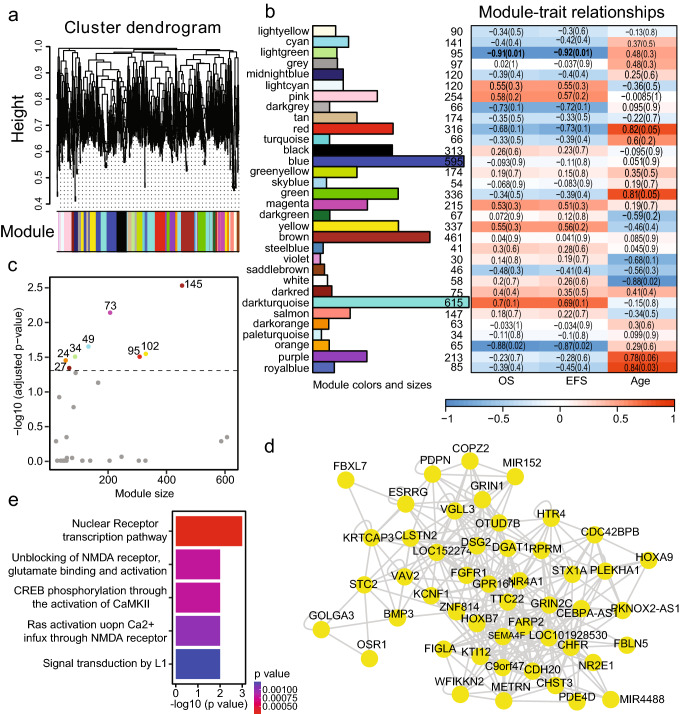
Co-epihet module identification and characterization. (**a**) Hierarchical clustering of genes with DEH loci in promoter regions based on gene co-epigenetic heterogeneity pattern across 6 AML patients. Each module is labeled by unique color. (**b**) In left panel, barplot visualizing the number of genes in each module. 31 modules were ultimately identified with sizes ranging from 30 to 615 genes. 97 genes were not assigned to any of 31 modules, and colored by grey. In right panel, correlation matrix of the module-trait relationship and corresponding p-values between the identified modules on the rows and clinical traits (survival time and ages) in 6 AML patients on the columns. The module-trait relationships are colored based on their correlation: red means a strong positive correlation, blue means a strong negative correlation. The lightgreen module significantly correlated with survival time of AML patients including overall survival (OS) time and event-free survival (EFS) time (p-value = 0.01). (**c**) Scatter plot showing the correlation between module sizes in x-axis and the corresponding − log10 (FDR adjusted p-values) in y-axis. Modules significantly enriched by DEGs are labeled the corresponding colors and the number of DEGs in the modules. Others are labeled grey. Otherwise, percentage of DEGs is showed in y-axis of scatter plot. (**d**) Module visualization of the lightgreen module using the cutoff 0.2 of correlation coefficient. (**e**) Pathway enrichment analysis for the lightgreen module (p-value < 0.05).

*epihet* is designed to identify differential epigenetic heterogeneity (DEH) loci in one cancer by comparing cancer with normal samples. Statistically significant DEH loci were selected using the absolute value of mean difference of epigenetic heterogeneity and multiple testing adjusted p-values calculated using t-test or permutation test. MA plot is employed to visualize DEH loci (Fig. 1c, see Supplementary Figs. S1e and S2e for PDR and Shannon entropy). Based on the identified DEH loci, *epihet* provides the tool for constructing co-epigenetic heterogeneity (co-epihet) network and performing network analysis to understand epigenetic mechanisms of cancers. The users can construct locus-level or gene-level co-epihet network. Here, *epihet* provides promoter, intron, extron, CpG shores and CpG island annotation files for gene annotation. The users can also download customized genome region in BED format from UCSC table browser which they are interested to associate genomic loci with genes, and create the GRanges objects *epihet* required by GemomicRanges Package^17^. Next*, epihet* identifies epigenetic modules for co-epihet network. Modules are labeled by different colors, here grey color is reserved to loci/genes which are not part of an epigenetic module (Fig. 2a). If the network nodes are DEH loci, *epihet* will return genes mapped by DEH loci in modules. Next, the number of genes in each co-epihet module is calculated (Fig. 2b). *epihet* also outputs the first principle component (PC1) of each module, which was calculated using epigenetic heterogeneity levels of all loci/genes within a module and represented the average epigenetic heterogeneity level of a module. When the users supply the clinical traits of patients, such as age, gender, survival time, *epihet* can identify clinically significant modules through evaluating the correlation between the PC1 of each module and clinical traits (Fig. 2b). Previous work has noted that promoter with high epigenetic heterogeneity showed low mean transcript levels, and high inter-sample transcriptional variation of the corresponding gene in chronic lymphocytic leukemia^8^. When comparing relapse to diagnostic AML samples, differentially expressed genes were significantly associated with promoters with a large epigenetic heterogeneity change^7^. Hence, the presence of epigenetic heterogeneity change at gene promoters resulted in greater tendency of the corresponding genes to show deregulated expression. Here, *epihet* allows the users to investigate the association between alterations in gene expression and the presence of DEH loci. *epihet* integrates co-epihet network with differentially expressed genes (DEGs) to determine modules significantly enriched by DEGs through hypergeometric test. In Fig. 2c, eight modules were significantly enriched by DEGs. The module sizes range from 63 to 461. The numbers of DEGs in modules range from 24 to 145. 95 genes of the lightgreen module is significantly enriched by 34 DEGs. Furthermore, modules can be visualized as networks showing the nodes and the edges between nodes. The user can specify the cutoff of the correlation coefficients to select the output of edges in the modules (Fig. 2d). *epihet* can also return the degree, centrality, betweenness and closeness of nodes in the module to help the users to understand the network topology property. Finally, *epihet* investigates the function of each module by performing pathway enrichment analysis using hypergeometric test. The users can specify cutoff for p-value to select significantly enriched pathways (Fig. 2e).

## Discussion

The new version *methclone v.*02 enables researchers to easily calculate a sample’s epiallele shift of each locus at two different stages and methylation pattern of each locus at each stage. Then, based on methylation pattern information from *methclone v.*02, R package *epihet* can calculate three types of intra-tumoral epigenetic heterogeneity for each locus in a sample. Additionally, *epihet* has functions to understand epigenetic mechanism in cancers. *epihet* can find the DEH loci by comparing cancer samples versus normal samples. The users can also select to compare different cancers or cancer subtypes. Biological networks and modules can show the collective behavior of groups of similar items, such as proteins, and their interactions with each other. *epihet* can further construct the co-epigenetic heterogeneity network (module) based on DEH loci or genes annotated by DEH loci. Finally, the users can perform all the analyses involved in *epihet* based on the customized epigenetic heterogeneity metrics. In summary, *epihet* fills a need of intra-tumoral epigenetic heterogeneity calculation tool and is valuable tool for intra-tumoral epigenetic heterogeneity study in cancers.

## Methods

### Epigenetic heterogeneity calculation

Epigenetic heterogeneity is calculated by considering the methylation state of a given locus (a group of adjacent four CpG sites). An epiallele is one of a number of alternative DNA methylation patterns of the same genetic locus. 4 CpGs on one locus can create 2^4^/16 different epialleles. Our tools can implement the four main methods for measuring epigenetic heterogeneity: proportion of discordant reads (PDR), Epipolymorphism, Shannon entropy and Delta entropy. The first three measures local epigenetic heterogeneity at a given locus, which can be calculated by our R package *epihet*. The difference of three methods is how variance of these patterns is calculated. Each method is built on slightly different assumptions and uses different equations. Additionally, each method has distinct advantages. The advantage of the PDR approach is its simplicity—it divides reads from the same locus into two categories: discordant vs concordant. Thus, it is straightforward to integrate PDR with other biological or genomic features. On the other hand, Epipolymorphism and Shannon entropy capture the information on all 16 possible epiallele patterns that can display at individual locus. Specifically, Epipolymorphism is a statistical measurement of variance. Epipolymorphism measures the probability of selecting two distinct patterns by randomly sampling. Whereas in information theory, Shannon entropy examines chaos in organized systems. Shannon entropy is designed to consider the proportion of all sixteen possible patterns together. Finally, Delta entropy evaluated the clonal dynamics of epialleles between different individuals or different stages within the same individual, which can be calculated by our tool *methclone v.02*.

#### Measure #1: Proportion of discordant reads

The proportion of discordant reads (PDR) is a measure of locally discordant DNA methylation^8^. A bisulfite sequencing read at a given locus was classified as a concordant read or a discordant read. Here, a concordant read is one that shows unmethylated or methylated state at all CpG sites of a given locus. A discordant read is one that shows varying methylated and unmethylated states at a given locus, such as one methylated cytosine followed by three unmethylated cytosines. PDR at each locus is defined as $\frac{Discordant\;read\;number}{{Total\;number\;of\;reads}}$, i.e. the proportion of discordant reads compared to the total number of reads from that locus.

#### Measure #2: Epipolymorphism

Epipolymorphism of a given locus in the cell population is defined as the probability that two epialleles randomly sampled from the locus differ from each other^14^. Epipolymorphism is calculated as $1 - \sum\nolimits_{i = 1}^{16} {p_{i}^{2} }$, where *p*_*i*_ is the fraction of each DNA methylation pattern *i* in the cell population.

#### Measure #3: Shannon entropy

Shannon entropy is defined as the chance that two randomly chosen epialleles (reads) have different methylated states of a given locus^15^. Shannon entropy of a given locus is calculated as $- \sum\nolimits_{i = 1}^{16} {p_{i} \log p_{i} }$, where *p*_i_ is the fraction of each DNA methylation pattern $i$ in the cell population.

#### Measure #4: Delta entropy

Different from three locally epigenetic heterogeneity measures above, Delta entropy (ΔS) considers the clonal dynamics of epialleles between different individuals, or within the same individual at different stages^13^. Briefly, the epiallele patterns of compositional changes between cancer patients and normal control samples were examined to calculate the combinatorial entropy change ($\Delta S$) of epialleles at each locus. Delta entropy quantifies the changes by using a composition entropy difference calculation. It ranges from no change (0) to maximum difference in entropy (− 144).

### Clustering analysis

*epihet* employed three methods to examine whether samples from one group form a biological meaningful cluster. First, *epihet* is used to cluster samples using hierarchical clustering analysis. The tool enables the users to select the distance metric between samples (e.g. “Euclidean”, “Manhattan” and so on) and cluster method used in the hierarchical clustering algorithm (e.g. “Ward’s method”, “complete method” and so on). *epihet* returns results as a heatmap with column annotation based on user-defined groups of the samples. Second, *epihet* can also perform Principal Component Analysis (PCA) on epigenetic heterogeneity matrix of samples. A scatter plot of the first two principal components could be created to show the highest variation through the data. Third, *epihet* allows the users to perform t-Distributed Stochastic Neighbor Embedding (t-SNE) analysis to map high-dimensional epigenetic heterogeneity matrix onto two dimensions while conserving the high-dimensional structure of the data. The users can also visualize the samples in a scatter plot based on the pairwise distances in high dimension. The plots can be colored based on user-defined groups of samples. Before the cluster analysis is performed, an epigenetic heterogeneity matrix has been formed, containing the samples and only those loci shared by at least a certain percentage of the samples. The users can specify epigenetic heterogeneity measure (PDR, Epipolymorphism, Shannon entropy or the customized metrics) through the argument ‘value’. In hierarchical clustering analysis, *epihet* has the argument to set the top of percentage of loci based on standard deviation to be used for analysis.

### Differential epigenetic heterogeneity loci identification

*epihet* identifies differential epigenetic heterogeneity (DEH) loci in the one cancer based on epigenetic heterogeneity mean difference of a locus between patients in cancer type and normal samples. To increase the statistical power, a two-stage approach was used that first filtered loci by a criterion independent of the statistical test^18^. *epihet* has the argument to set the absolute value of mean difference of epigenetic heterogeneity values of a given locus between two groups, such as cancer and normal control samples to filter some loci for further analysis. For PDR and Epipolymorphism, we designed *epihet* to implement t-test for determining significance of differential epigenetic heterogeneity across all the loci. Significantly differential Shannon entropy was assessed using permutation test^19^. The default number of permutation tests to be implemented is set to 1000. Specifically, given a locus, there are two lists of Shannon entropy for *n*_1_ cancer samples and *n*_2_ normal samples. First, we calculated the difference of Shannon entropy between cancer and normal samples. Then, we generated a new list *A* containing all *n*_1_ + *n*_2_ Shannon entropy from the two lists. Next we randomly permute the elements of *A*, then recalculate differential Shannon entropy, where the first *n*_1_ elements of *A* as Shannon entropy of cancer samples, the last *n*_2_ elements of *A* as Shannon entropy of normal samples. Finally, p-value was defined as the proportion of the random differential Shannon entropy that are larger than or equal to the original differential Shannon entropy. Additionally, the users can select one of t-test and permutation test to identify DEH loci for the customized metrics through the argument ‘permutationtest’. Statistically significant DEH loci were selected based on multiple testing adjusted p-values and the absolute value of mean difference of epigenetic heterogeneity. The users can define increased/decreased DEH loci based on the sign of the difference of mean epigenetic heterogeneity between cancer patients and normal control samples.

### Co-epigenetic heterogeneity network construction

#### Co-epigenetic heterogeneity network and module construction

Biological network has been widely used to describe interactions between biological entities of interest. Co-methylation network and epigenetic modules play important roles in understanding epigenetic mechanisms of cancers^20,21^. *epihet* enables the users to construct co-epigenetic heterogeneity (co-epihet) network based on the DEH loci using the WGCNA R package^22^. We have two options to be employed to construct co-epihet network. The first option is to measure the correlation of epigenetic heterogeneity between any two DEH loci and construct locus-level co-epihet network. The second option is to measure the correlation of mean epigenetic heterogeneity between any two genes containing DEH loci within genomic region and construct gene-level co-epihet network. Allowed correlation methods are Pearson and biweight midcorrelation. Then, *epihet* identified co-epihet modules using hierarchical clustering, in which loci/genes were highly correlated in their epigenetic heterogeneity levels.

#### Module annotation

Firstly, Reactome pathway enrichment analysis were performed through a hypergeometric test. The enrichment analyses were performed using the ‘ReactomePA’ R package^23^. Then, *epihet* annotated co-epihet modules by the differentially expressed genes (DEGs), which the users can provide. *epihet* uses a hypergeometric test to identify the significantly enriched modules by DEGs. If no module is significantly enriched by DEGs, *epihet* returns the modules which contain DEGs. *epihet* draws scatter plot that visualizes the distribution of adjusted p-values for modules significantly enriched by DEGs or the distribution of percentage of DEGs for modules annotated by DEGs.

## Supplementary Information

Supplementary Figures.
