# Supplementary material for: epihet for intra-tumoral epigenetic heterogeneity analysis and visualization
Source: Sci Rep. 2021 Jan 11;11:376. doi: 10.1038/s41598-020-79627-x (PMC7801679; doi:10.1038/s41598-020-79627-x)
Supplement: Supplementary file 1 — Supplementary Figures. [file 41598_2020_79627_MOESM1_ESM.docx]

***epihet* for intra-tumoral epigenetic heterogeneity analysis and visualization**

Xiaowen Chen^1^, Haitham Ashoor^1^, Ryan Musich^1^, Jiahui Wang^1^, Mingsheng Zhang^1^, Chao Zhang^2^, Mingyang Lu^3^, Sheng Li^1, 4, 5, 6*^

^1^The Jackson Laboratory for Genomic Medicine, 10 Discovery Drive, Farmington, CT 06032-2374, USA

^2^Weill Cornell Medicine, New York, NY, USA

^3^The Jackson Laboratory for Mammalian Genetics, Bar Harbor, ME, USA

^4^The Jackson Laboratory Cancer Center, Bar Harbor, ME, USA

^5^Department of Genetics and Genome Sciences, University of Connecticut School of Medicine, Farmington, CT, USA

^6^Department of Computer Science and Engineering, University of Connecticut, Storrs, CT, USA

*Correspondence: [sheng.li@jax.org](mailto:sheng.li@jax.org)

**Supplemental Information**

**Figure S1. Related to Figure 1.**

*epihet* compares PDR difference and identifies DEH loci using the same samples in Figure 1C.

(a) Boxplot shows PDR of patients with CEBPA-sil mutation are higher than normal samples.

(b) A two-dimensional principal components analysis of 6 AML patients and 14 normal samples clustered into 2 groups.

(c) Heatmap of PDR value based on the most variant 5% loci across 6 AML patients and 14 normal samples using hierarchical clustering analysis.

(d) t-SNE visualizations of 6 AML patients and 14 normal samples clustered into 2 groups.

(e) MA plot showing the DEH loci between AML patients and normal samples (absolute value of mean epigenetic heterogeneity difference > 0.2, FDR adjusted p value <0.05), which are highlighted in red.

**Figure S2. Related to Figure 1.**

*epihet* compares Shannon entropy difference and identifies DEH loci using the same samples in Figure 1C.

(a) Boxplot shows Shannon entropy of patients with CEBPA-sil mutation are higher than normal samples.

(b) A two-dimensional principal components analysis of 6 AML patients and 14 normal samples clustered into 2 groups.

(c) Heatmap of Shannon entropy value based on the most variant 5% loci across 6 AML patients and 14 normal samples using hierarchical clustering analysis.

(d) t-SNE visualizations of 6 AML patients and 14 normal samples clustered into 2 groups.

(e) MA plot showing the DEH loci between AML patients and normal samples (absolute value of mean epigenetic heterogeneity difference > 0.2, FDR adjusted p value <0.05), which are highlighted in red.
